# Impact on Spine Surgery during the First Two Years of COVID-19 Pandemic: A Nationwide Study in South Korea

**DOI:** 10.3390/jcm12124155

**Published:** 2023-06-20

**Authors:** Chang-Min Ha, Yunjin Nam, Sungjoon Lee, Se-Jun Park, Sun-Ho Lee, Eun-Sang Kim

**Affiliations:** 1 Department of Neurosurgery, Samsung Medical Center, Sungkyunkwan University School of Medicine, 81, Irwon-ro, Gangnam-gu, Seoul 06351, Republic of Korea; hcm8640@naver.com (C.-M.H.); potata98@gmail.com (S.L.); sobotta72@hotmail.com (S.-H.L.); 2 Department of Orthopedic Surgery, Korea University Guro Hospital, Korea University College of Medicine, 148, Gurodong-ro, Guro-gu, Seoul 08308, Republic of Korea; nam.yunjin@gmail.com; 3 Department of Orthopedic Surgery, Samsung Medical Center, Sungkyunkwan University School of Medicine, 81, Irwon-ro, Gangnam-gu, Seoul 06351, Republic of Korea; sejunos.park@samsung.com

**Keywords:** COVID-19, nationwide data, spine, spine surgery

## Abstract

Since December 2019, the novel coronavirus (COVID-19) has infected people worldwide. Owing to its rapid spread, elective surgeries, including spine surgery, have been re-scheduled. We analyzed nationwide data to investigate changes in the volume of spine surgery during the first two years of the pandemic. Nationwide data from January 2016 to December 2021 were obtained. We compared the total number of patients who underwent spine surgery and related medical expenses before and during the COVID-19 pandemic. In February and September, the number of patients was significantly smaller compared to January and August, respectively. Despite the pandemic, the proportion of patients undergoing spine surgery for degenerative diseases in 2021 was the highest. In contrast, the proportions of patients undergoing spine surgery for tumors showed a continuous decrease from 2019 to 2021. Although the number of spine surgeries performed at tertiary hospitals was lowest in 2020, it was not significantly smaller than that in 2019.The number of patients who underwent spine surgery in March 2020, during the first outbreak, decreased compared to the previous month, which differed from the trend observed in the pre-COVID-19 period. However, as the pandemic continues, the impact of COVID-19 on spine surgery has become less evident.

## 1. Introduction

Coronaviruses are common among humans and cause respiratory infections [1,2]. Six coronavirus species have been known to cause human diseases, including severe acute respiratory syndrome coronavirus (SARS-CoV) and Middle East respiratory syndrome coronavirus (MERS-CoV) [3]. In contrast to the other four coronaviruses, SARS-CoV and MERS-CoV led to serious outbreaks in 2002 and 2003 in China and in 2012 and 2013 in the Middle East, respectively [4,5].

In December 2019, a number of patients with pneumonia of unknown etiology were reported from Wuhan, People’s Republic of China, and a novel coronavirus was found to be the etiology [3]. On 11 February 2020, the World Health Organization (WHO) announced that the disease caused by the novel coronavirus would be named COVID-19 [6]. One month later, on 11 March 2020, the WHO declared that COVID-19 should be characterized as a pandemic, considering that more than 118,000 COVID-19 patients were diagnosed among 114 different countries and more than 4000 people died [7].

Elective surgeries have been recommended to be rescheduled during the pandemic, owing to limited resources and the COVID-19 burden [8]. Moreover, a consensus regarding COVID-19 patients suggests that elective surgeries should not be scheduled within seven weeks of a COVID-19 diagnosis, considering the risk of postoperative morbidity or mortality related to COVID-19 [9]. Spine surgery is also expected to be affected by the pandemic, since spine surgery is one of the top 10 major surgeries in South Korea in terms of both the number of patients and the medical expenses related to the surgery [10]. Thus, identifying whether there have been changes in spine surgery during the COVID-19 pandemic era will be helpful in rearranging medical and human resources and may offer insight into dealing with other pandemics in the future.

To our knowledge, no study to date has identified the impact of COVID-19 on spine surgery by including two years of nationwide data since the appearance of COVID-19 in the analysis. This study aimed to investigate the influence of COVID-19 on spine surgery in South Korea by comparing the number of patients who underwent spine surgery and medical expenses related to spine surgery during the COVID-19 pandemic with those before the COVID-19 pandemic.

## 2. Materials and Methods

### 2.1. Data Collection

All South Korean citizens receive the benefits of the South Korean National Health Insurance System (NHIS). Moreover, nationwide data on disease and services, including procedures and operations, are structurally coded and registered in the South Korean Health Insurance Review and Assessment Service (HIRA) database, thus enabling the population-based studies [11,12].

The data used in this study were obtained from the HIRA database. Anonymized and statistically processed data were acquired by appropriate and systematic request via the Public Data Portal (https://www.data.go.kr/en/index.do, accessed on 1 December 2022), operated by South Korean Ministry of the Interior and Safety. If one of the 48 demand codes related to spinal surgery was claimed to the NHIS, it was defined as ‘spine surgery’. The disease codes used in this database were standardized according to the Korean Classification of Disease, eighth version, which followed the International Classification of Diseases, 10th Edition (ICD-10) [11,12,13].

### 2.2. Study Design

Ethical approval was waived by the Institutional Review Board (IRB) of Samsung Medical Center since this study used de-identified public data (IRB number: 2022-12-033). The total number of patients who underwent spine surgery and the medical expenses related to spine surgery from January 2016 to December 2021 were obtained on a monthly basis. As the first report of COVID-19 in South Korea was made on 20 January 2020, the ‘pre-COVID-19’ group was defined as the period from January 2016 to December 2019, whereas the ‘during COVID-19’ group was defined as the period from January 2020 to December 2021.

We defined the ‘during COVID-19’ group starting from January 2020 due to the following reasons. Firstly, although the first outbreak in South Korea occurred in March 2020, the government responded immediately after detecting the first COVID-19 patient on 20 January 2020. Secondly, based on the lessons learned from the impact of MERS-CoV on South Korea, medical institutions implemented measures to prevent the spread of COVID-19 within their facilities. Thirdly, due to the real-time reporting of COVID-19 spread by the mass media, many people were already aware of COVID-19 even before the first outbreak. Therefore, January 2020 was chosen as the starting point, as both individual behavior and the practices of medical institutions were already influenced by COVID-19 even before the official declaration of the pandemic or the first outbreak in South Korea.

The total number of patients and medical expenses per year were obtained by adding the number and medical expenses, respectively, of the corresponding 12 months. Medical expenses were defined as the sum of the co-payment paid by the patient and the insurance benefit paid by the NHIS. The insurance benefit, paid by the NHIS, represents the amount of reimbursement provided by the insurer (NHIS) to the healthcare provider. This amount is determined based on the total medical cost as reviewed by HIRA (excluding the co-payment paid by the patient).

The monthly values were further subdivided based on disease category or provider type. Diseases were categorized into the following five categories using disease codes: degenerative diseases (M codes, excluding M00, M01, M03, M46.2, M46.3, M46.4, M46.5, M49.0, M49.1, M49.2, M49.3, M60, M71.0, M71.1, and M86), trauma (S codes), tumor (C and D codes), infection (G06, M00, M01, M03, M46.2, M46.3, M46.4, M46.5, M49.0, M49.1, M49.2, M49.3, M60, M71.0, M71.1, M86, T81, T84, and T85), and miscellaneous diseases (disease codes that did not fall under the categories of degenerative diseases, trauma, tumor, or infection).

Provider types were classified into the following four categories based on the number of hospital beds, the number of specialized departments, and whether they provide inpatient or outpatient care: tertiary hospitals (20 or more specialized departments with 300 or more beds), general hospital (7 or more specialized departments with 100 or more beds), hospitals (institutions with 30 or more beds, regardless of the number of specialized departments, and clinics (institutions primarily focused on outpatient care). The monthly and yearly volumes of spine surgery between the two groups were compared. Further subgroup analysis was also performed according to disease category or provider type. During the study period, there were four COVID-19 outbreaks in South Korea [14]. The first outbreak occurred in March 2020, followed by the second outbreak in August 2020, the third outbreak in December 2020, and the fourth outbreak in September 2021. The impact of each outbreak on the volume of spine surgery was identified by reviewing the volumes of spine surgery before and after every outbreak.

### 2.3. Statistical Analysis

Statistical analyses were performed using SPSS^®^ Statistics 27 (IBM Co., Armonk, NY, USA). Continuous variables are presented as actual numbers or mean ± standard deviation. Categorical variables are presented as frequencies. Categorical variables were compared using the Chi-square test, while continuous variables were compared using the Mann–Whitney test and Kruskal–Wallis test. A two-tailed *p* < 0.05 was considered statistically significant.

## 3. Results

### 3.1. Number of Patients Who Received Spine Surgery

The mean number of monthly patients who received spine surgery in 2016, 2017, 2018, 2019, 2020, and 2021 were 13,391.2 ± 786.1, 13,894.8 ± 795.4, 14,404.4 ± 1363.4, 14,934.7 ± 1078.3, 15,264.6 ± 1280.0, and 16,426.1 ± 1086.5, respectively (*p* < 0.001) (Table 1). In the pre-COVID-19 group, 14,156.3 ± 1156.4 patients received spine surgery per month, in contrast to 15,845.6 ± 1303.9 patients in the during COVID-19 group (*p* < 0.001).

The monthly trend of the mean number of patients who received spine surgery showed that those in February and September were the lowest within a year (12,930.3 ± 736.8 and 13,476.8±1255.1, respectively). However, only the number of patients who received spine surgery in February was significantly lower than that per month (*p* = 0.002). The number of patients who received spine surgery in February was compared with those of the months before and after, and was significantly smaller than those in both January (12,930.3 ± 736.8 vs. 15,916.7 ± 1017.3, *p* < 0.001) and March (12,930.3 ± 736.8 vs. 14,664.0 ± 1683.7, *p* = 0.043).

The number of patients who received spine surgery in September was also compared with those in the months before and after. The number of patients who received spine surgery in September was significantly smaller than that in August (13,476.8 ± 1255.1 vs. 15,234.7 ± 911.6, *p* = 0.020), but not significantly smaller than that in October (13,476.8 ± 1255.1 vs. 14,565.5 ± 1374.6, *p* = 0.182).

### 3.2. Medical Expenses Related to Spine Surgery

The mean medical expenses related to spine surgery per month in 2016, 2017, 2018, 2019, 2020, and 2021 were 9342.3 ± 626.3, 9969.0 ± 596.5, 11,484.8 ± 1185.6, 12,665.1 ± 962.6, 14,044.7 ± 1166.7, and 15,402.1 ± 1122.1 million KRW, respectively (*p* < 0.001) (Table 2). In the pre-COVID-19 group, the medical expenses related to spine surgery were 10,865.3 ± 1563.1 million KRW compared to 14,723.4 ± 1316.8 million KRW in the during COVID-19 group (*p* < 0.001).

The monthly trend of medical expenses related to spine surgery also showed that those in February and September were the lowest within a year (10,766.4 ± 1904.6 and 10,918.4 ± 2122.4 million KRW, respectively). However, the medical expenses related to spine surgery in these months were not significantly lower than those per month (*p* = 0.135 and *p* = 0.214, respectively). The medical expenses related to spine surgery in February were compared to those of January (10,766.4 ± 1904.6 vs. 13,337.7 ± 2296.8 million KRW) and March (10,766.4 ± 1904.6 vs. 12,375.4 ± 2756.4 million KRW), respectively, but no significant difference was observed (*p* = 0.061 and *p* = 0.267, respectively).

In addition, the medical expenses related to spine surgery in September were further compared with those in August (10,918.4 ± 2122.4 vs. 12,392.3 ± 2110.9 million KRW) and October (10,918.4 ± 2122.4 vs. 12,020.9 ± 2423.9 million KRW), respectively. No significant difference was observed (*p* = 0.256 and *p* = 0.421, respectively).

### 3.3. Number of Patients Who Received Spine Surgery by Disease Category or Provider Type

**Figure 1:**
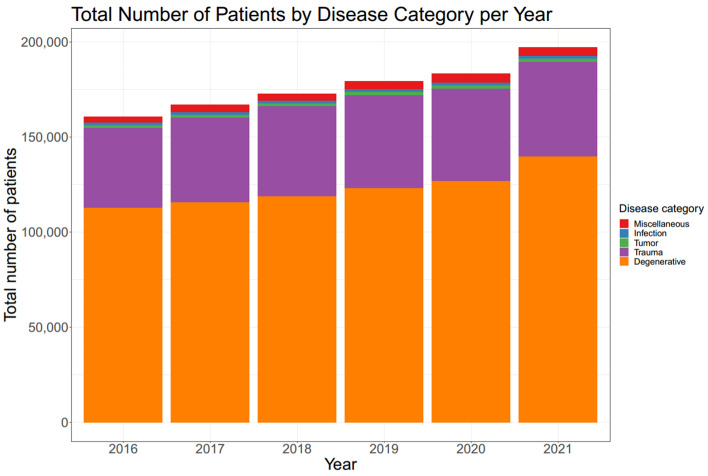
Total number of patients by disease category per year. Number of patients who received spine surgery each year is shown. The numbers continuously increased from 2016 to 2021. Degenerative disease was the most common disease category.

**Figure 2:**
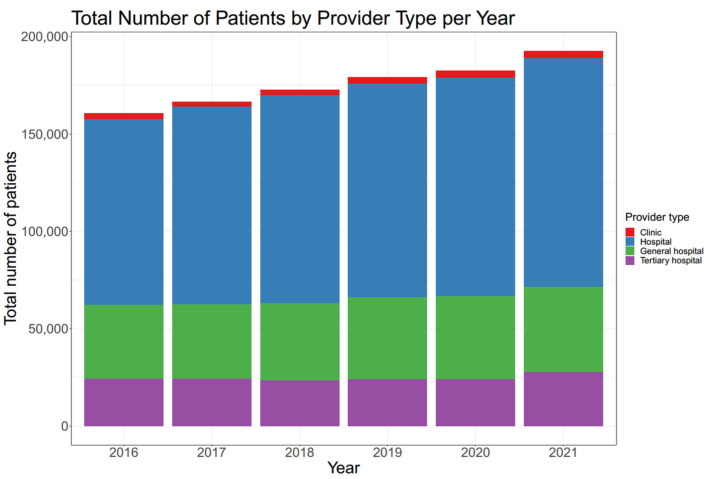
Total number of patients by provider type per year. The number of patients who received spine surgery each year was subdivided according to provider type, and hospital was the most common provider.

**Figure 3:**
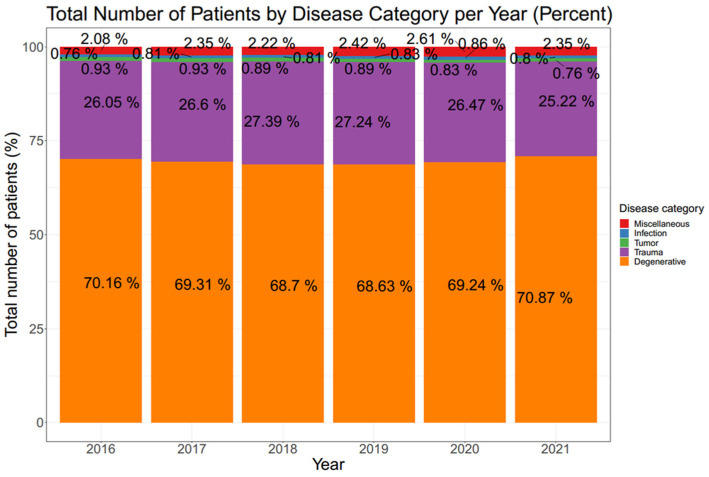
Total number of patients by disease category per year (percent). Proportions of each disease category every year are shown. Infection and tumor each accounted for less than 1% of cases.

The number of patients who underwent spine surgery exhibited an annual increase from 2016 to 2021 (Table 1; Figure 1 and Figure 2). These cases were further analyzed based on disease category, revealing that degenerative disease was the most common category, ranging from 68.63% to 70.87%. Trauma accounted for the second highest proportion, ranging from 25.22% to 27.39%. Infection and tumor cases each represented less than 1% of the total. This trend was observed consistently in each year analyzed throughout the study period (Figure 3).

In 2021, the proportion of patients undergoing spine surgery for degenerative diseases was the highest during the study period, accounting for 70.87%. Conversely, the proportion of patients undergoing spine surgery for trauma in the same year was the lowest, at 25.22%. Additionally, when considering the entire study period, the proportion of patients undergoing spine surgery for degenerative diseases ranked second highest, accounting for 70.16% of cases, while the proportion for trauma ranked second lowest, at 26.05% in 2016. Both of these proportions were found to be significantly different compared to those in 2021 (70.16% vs. 70.87%, *p* < 0.001 and 26.05% vs. 25.22%, *p* < 0.001).

The proportions of patients undergoing spine surgery for tumors in 2019, 2020, and 2021 were 0.89%, 0.83%, and 0.76%, respectively. The proportions of patients undergoing spine surgery for tumors in 2019 and 2020 were not significantly different (*p* = 0.087), but the proportion in 2021 was significantly smaller than that in 2019 (*p* < 0.001). On the other hand, the proportions of patients undergoing spine surgery for infection or miscellaneous diseases in 2020 or 2021 did not show a significant decrease or increase compared to those from 2016 to 2019, as shown in Figure 3.

**Figure 4:**
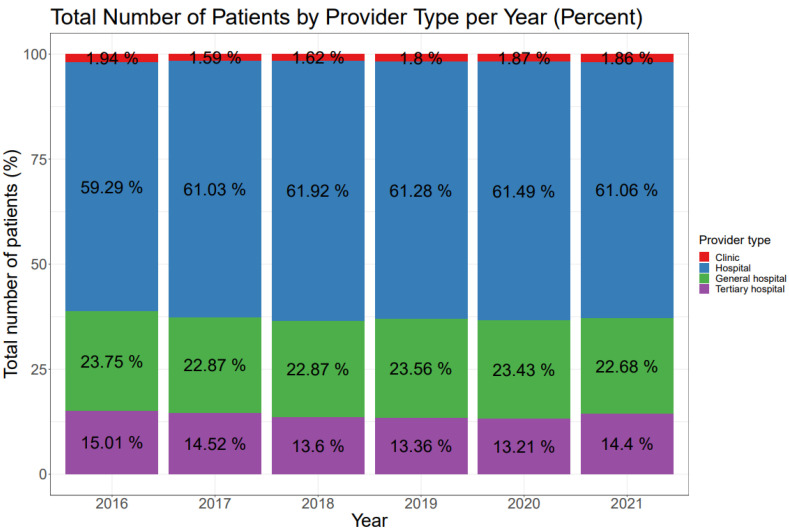
Total number of patients by provider type per year (percent). Proportions of each provider type every year are shown. The proportion of spine surgery carried out in tertiary hospital was the lowest in 2020 during the study period.

In addition, the number of patients who received spine surgery was analyzed according to the provider type. Spine surgeries were most frequently performed in hospital (range 59.29–61.92%), followed by general hospital (range 22.68–23.75%) and tertiary hospital (range 13.21–15.01%). The proportion of spine surgeries performed in tertiary hospital was the lowest in 2020, but it was not significantly smaller than that in 2019 (*p* = 0.198). However, the proportion of spine surgeries performed in tertiary hospital in 2021 significantly increased compared to that in 2020 (*p* < 0.001), as shown in Figure 4.

### 3.4. Volume of Spine Surgery during COVID-19 Outbreaks

**Figure 5:**
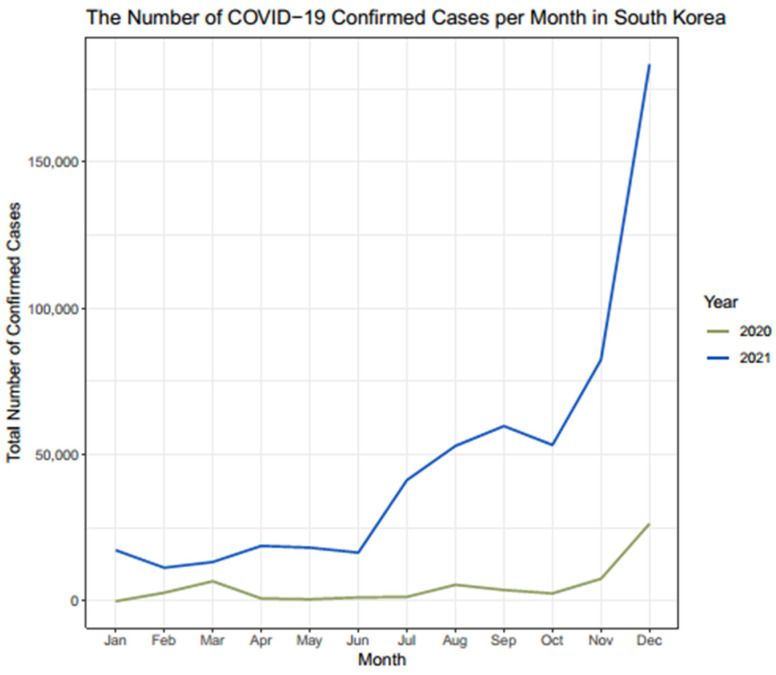
Number of COVID-19-confirmed cases per month in South Korea. The number of COVID-19-confirmed cases is shown on a monthly basis. Four outbreaks were observed in South Korea during the study period. The first outbreak occurred around March 2020. The second and third outbreaks took place around August 2020 and December 2020, respectively. The fourth outbreak, which was the longest in the study period, occurred around September 2021.

**Figure 6:**
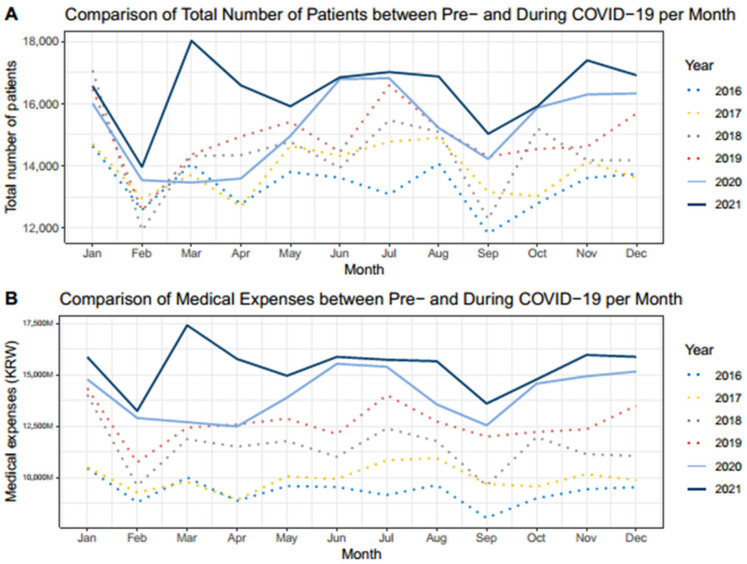
Comparison of volumes of spine surgery between pre- and during COVID-19 periods. Both the number of patients who received spine surgery and medical expenses related to spine surgery were lower in February and September compared to those of other months. (**A**) The number of patients who received spine surgery in March 2020 was lower than that of February 2020, in contrast to the trend in previous years. The number of spine surgeries performed in March was the lowest in 2020 in the study period. (**B**) Medical expenses related to spine surgery in March 2020 also decreased compared to those of February 2020. However, medical expenses were not the lowest in 2020 in the study period.

During the study period, there were four COVID-19 outbreaks in South Korea (Figure 5) [14]. The first COVID-19 outbreak in South Korea occurred in March 2020. Prior to 2020, the number of patients who received spine surgery and medical expenses related to spine surgery in March were greater than those in February. However, those in March 2020 were smaller than those in February 2020 (13,470 vs. 13,548 patients; 12,703.2 vs. 12,905.0 million KRW, respectively). Meanwhile, an increasing trend in the volume of spine surgery from February to March was identified again in 2021 (Figure 6).

The second and third outbreaks occurred in August 2020 and December 2020, respectively. The number of patients who received spine surgery and medical expenses related to spine surgery in September 2020 were both at a minimum compared to those in the months before and after. The volume of spine surgeries in December 2020 was similar to that in November 2020 and January 2021. These trends were observed around the two outbreaks, and did not differ from the trends of the same period in previous years.

The fourth outbreak occurred in September 2021; however, the duration of the outbreak was longer than that of the previous three outbreaks. Nevertheless, the monthly trend in the volume of spine surgery around September 2021 did not differ from the monthly trend in the same period among the previous years (i.e., the volume of spine surgery was smaller in September compare to that of August and October; see Table 1 and Figure 6).

## 4. Discussion

In this study, the monthly volume of spine surgery consistently increased on an annual basis throughout the study period. Degenerative disease was identified as the most common category, followed by trauma. In 2021, the proportion of patients undergoing spine surgery for degenerative diseases was the highest, while the proportion of trauma cases was the lowest (both *p* < 0.001). The proportion of spine surgeries performed for tumor cases in 2021 was significantly lower compared to the pre-COVID period. Additionally, spine surgeries were primarily performed in hospitals, followed by general hospitals and tertiary hospitals. The proportion of spine surgeries performed in tertiary hospitals was the lowest in 2020, but significantly increased in 2021 (*p* < 0.001).

As of 1 January 2022, the novel coronavirus had infected more than 289,000,000 patients and caused more than 5,400,000 deaths worldwide [15]. Over 635,000 infections and 5600 deaths have been reported in South Korea since the first report of COVID-19 on 20 January 2020 [14]. COVID-19 is the first pandemic caused by a coronavirus in human history [7], but unfortunately, this pandemic is still ongoing and has significantly affected our daily lives. COVID-19 is a social phenomenon, beyond a rapidly spreading upper respiratory infection [16]. Wearing a mask, self-isolation, and physical distancing have become ordinary habits [17].

During the pandemic, the governmental guidelines in South Korea primarily focused on ‘physical distancing’ as a preventive measure to inhibit contact between people and prevent the spread of COVID-19. During the first COVID-19 outbreak in March 2020, it was the first time that the government advised people to practice physical distancing. During the second outbreak in August 2020, restrictions on movement to other areas were advised, and religious facilities were closed. In December 2020, during the third outbreak, a ban on gatherings of more than five people was enforced in the metropolitan area, including Seoul. In the fourth and most severe outbreak within the study period, even tighter restrictions were put in place, including limitations on the use of public facilities by individuals who had not been vaccinated against COVID-19 or did not have a PCR-negative certificate.

There were no specific governmental guidelines for medical personnel in medical institutions. Instead, the guidelines primarily focused on regulations that should be followed in everyday life, as mentioned previously. However, nearly all medical institutions implemented their own standards, which required mandatory COVID-19 PCR testing before patient admission. Patients who tested negative underwent surgery with surgeons wearing dental masks, while only PCR-positive patients who could not delay urgent surgeries underwent surgery with N95-wearing surgeons in specialized facilities that included negative pressure environments.

For PCR-positive patients, elective surgeries were postponed and rescheduled. However, emergent or urgent surgeries for PCR-positive patients were performed according to the guidelines provided by the Korean Neurosurgical Society (not available in English, but similar to the triaging system outlined by Jain et al. [18]). Specifically, emergent surgeries were performed for patients who had progressive or severe neurological deficits due to neurological compression or spinal instability at risk of causing neurological injury. Urgent surgeries were performed for patients with myelopathy due to spinal stenosis with recent progression, or spinal infections (such as discitis, osteomyelitis, or epidural abscess) that failed to respond to medical management. Nonetheless, the decision to proceed with surgery was ultimately based on the individual patient’s condition and the clinical judgment of the surgeon, following the aforementioned guidelines.

The impact of COVID-19 is broad and substantial; thus, spine surgery cannot be an exception to the COVID-19 pandemic. Since the early period of the pandemic, researchers have discussed spine surgery and COVID-19 [19,20,21]. The influence of COVID-19 on spine surgery in specific regions has also been reported [22,23]. Moreover, Louie et al. conducted the first international study to assess the effects of COVID-19 among spine surgeons [24], and a follow-up study by Barajas et al. showed the responses of spine surgeons throughout one year [25]. A scoring system for the triage of spine surgeries due to the limited medical and human resources was also suggested by two individual studies [18,26]. Furthermore, COVID-19 has forced the adoption of ‘virtual medicine’ in practice [25,27], and affected the training conditions for residents [21,28].

Some authors have discussed whether the number of spine surgeries has been affected by COVID-19. Lee et al. showed that the number of elective spine surgeries in a single institution decreased during the COVID-19 pandemic compared to the same period over the past two years [29]. Likewise, similar results were presented by Riley and Verma [30]. Different conclusions were suggested by other researchers. Ham et al. demonstrated that the number of hospital visits significantly decreased, but the number of spine surgeries was not actually affected [16]. Ghermandi et al. reported that the number of spine surgeries increased from March 2020 to May 2020 compared to that of the same period in 2019 [18].

Prior research has mainly discussed the impact of COVID-19 on the number of spine surgeries performed in a single institution. Two other studies have demonstrated the nationwide impact of spine surgery due to COVID-19, but the study period was limited mainly to the first year of the COVID-19 pandemic [31,32]. The study by Idrizi et al. [31] was the only study performed in a country other than South Korea. However, they specifically focused on elective cervical spine surgery in the United States during the first year of the pandemic. They demonstrated a decrease in the volume of spine surgery during the second quarter of 2020, which aligns with our study findings of a significant decrease in the volume of spine surgery in March 2020 (Figure 6). However, unlike our study, their findings showed that the volume of elective surgery did not return to the pre-pandemic baseline. In contrast, our analysis of the volume of spine surgery during the first two years of the pandemic demonstrated that the volume eventually returned to the pre-pandemic levels.

Due to the ongoing crisis, national data for the first two years of the COVID-19 pandemic were analyzed in this study. Interestingly, February and September were the lowest points on a yearly basis in both groups. This may be because of the unique situations in South Korea. Education-related reasons, such as avoiding children’s winter vacation in February when scheduling surgeries, may have contributed to this result. In addition, social and cultural causes, such as Lunar New Year’s Day (usually in February) and Korean Thanksgiving Day (usually in September), might be another reason.

Meanwhile, four outbreaks occurred in South Korea during the study period. The first outbreak occurred in March 2020, and the volume of spine surgeries decreased compared to that in the previous month. This change was not observed in the pre-COVID-19 group. However, the daily number of confirmed COVID-19 cases during the first outbreak was relatively smaller than that of the later outbreaks [14]. Thus, the decreased volume of spine surgery during the first outbreak was not directly related to the number of confirmed cases, but was rather affected by other factors. This includes preemptive actions during the initial period of the pandemic, such as a longer isolation period of 14 days, broad criteria for self-isolation, voluntary shutdown of medical institutions visited by confirmed patients, or strict standards for scheduling surgeries.

The second outbreak occurred in August 2020, but the period partially overlapped with the low points stated above, and its effect was uncertain. The third outbreak followed the second outbreak and peaked in December 2020. The trend in the number of patients who received spine surgery and medical expenses related to spine surgery during the third outbreak showed no difference from that of the same period in previous years. The fourth outbreak, which was the longest outbreak in the study period, was the only outbreak in 2021. It persisted for nearly seven months, from June 2021 to December 2021. The peak was found to be around September 2021, and the volume of spine surgery decreased compared to that in August, as in previous years.

However, this study had several limitations. It was designed as a retrospective study, and the data used did not contain other information, such as patient demographics, diagnosis, or type of surgery. These factors may have affected the results. In addition, changes in the volume of spine surgery can be due to individual needs or perceptions regarding spine surgery or even public health awareness. Nevertheless, investigating the first two years of the COVID-19 pandemic may be helpful in preparing for future pandemics.

In contrast to the ongoing nature of the COVID-19 pandemic, the volume of spine surgery in South Korea only decreased during the first outbreak in March 2020. By May or June 2020, the volume of spine surgery had returned to the pre-COVID-19 baseline levels, indicating that the impact on the volume of spine surgery was not evident after the first outbreak. This suggests that individual surgeons or institutions should not only respond appropriately to COVID-19-related policies, but also provide optimal surgical treatment to patients with spinal diseases. It is essential to maintain the ability to respond to new pandemics while upholding the usual standard of care, as the emergence of future pandemics is inevitable.

Additionally, in the subgroup analysis, degenerative disease remained the most common category throughout the entire study period. Interestingly, the proportion of degenerative disease cases, which are typically considered less urgent compared to trauma or tumor cases, even increased significantly during the COVID-19 period, from 69.24% in 2020 to 70.87% in 2021. On the other hand, the proportion of patients undergoing spine surgery for tumors showed a continuous decrease from 2019 to 2021. This decline may be a consequence of the postponement of non-emergent or non-urgent tumor surgeries during the pandemic.

The postponement of non-emergent or non-urgent surgeries for conditions other than degenerative diseases during the pandemic may have allowed degenerative disease cases to make up a higher proportion of spine surgeries, even during the pandemic. Tumor cases typically require more extensive medical resources within institutions compared to patients with degenerative diseases. As a result, there may have been a tendency to delay tumor surgeries during the COVID-19 pandemic, which could contribute to the decreasing proportions of tumor cases in spine surgeries.

Furthermore, the proportion of spine surgeries performed in tertiary hospitals was the lowest in 2020, but showed a significant increase in 2021. This finding may be explained by the considerable burden of COVID-19 experienced by tertiary hospitals during the initial year of the pandemic. The high demands for treating COVID-19 patients could have reduced the capacity for elective spine surgeries in these hospitals. Overall, these observations reflect the burden faced by healthcare institutions during the COVID-19 pandemic, as they had to manage both the treatment of COVID-19 patients and the performance of spine surgeries.

## 5. Conclusions

During the early period of the COVID-19 pandemic, a significant decrease in the number of patients who received spine surgery and medical expenses related to spine surgery was observed. Among the four outbreaks in South Korea, the volume of spine surgeries was only affected during the first outbreak. The trend in the monthly volume of spine surgery in 2021 was similar to that of the pre-COVID-19 period. As the pandemic continues, the volume of spine surgery may restore ordinary levels; therefore, spine surgeons should be thoroughly prepared for patients with spinal disease.

## Figures and Tables

**Table 1:** Total number of patients who received spine surgeries from January 2016 to December 2021.

Year/Month	2016	2017	2018	2019	2020	2021	Mean ± SD *	*p*-Value
January	14,644	14,712	17,076	16,477	16,014	16,577	15,916.7 ± 1017.3	
February	12,592	12,966	11,931	12,571	13,548	13,974	12,930.3 ± 736.8	
March	14,074	13,722	14,324	14,367	13,470	18,027	14,664.0 ± 1683.7	
April	12,780	12,701	14,350	14,950	13,599	16,592	14,162.0 ± 1478.0	
May	13,810	14,652	14,781	15,421	14,968	15,920	14,925.3 ± 717.8	
June	13,625	14,328	13,946	14,453	16,792	16,851	14,999.2 ± 1441.5	
July	13,092	14,779	15,474	16,590	16,823	17,021	15,629.8 ± 1514.6	
August	14,080	14,910	15,099	15,218	15,224	16,877	15,234.7 ± 911.6	
September	11,834	13,168	12,292	14,305	14,226	15,036	13,476.8 ± 1255.1	
October	12,798	13,035	15,208	14,550	15,877	15,925	14,565.5 ± 1374.6	
November	13,617	14,140	14,185	14,623	16,299	17,397	15,043.5 ± 1475.1	
December	13,748	13,625	14,187	15,691	16,335	16,916	15,083.7 ± 1414.8	
Mean ± SD *	13,391.2 ± 786.1	13,894.8 ± 795.4	14,404.4 ± 1363.4	14,934.7 ± 1078.3	15,264.6 ± 1280.0	16,426.1 ± 1086.5	14,719.3 ± 1441.8	<0.001 ^†^

Values are presented as exact number or mean ± standard deviation; * SD, standard deviation; ^†^ *p*-value < 0.05, statistically significant.

**Table 2:** Medical expenses related to spine surgery from January 2016 to December 2021.

Year/Month	2016	2017	2018	2019	2020	2021	Mean ± SD *	*p*-Value
January	10,462.6	10,504.2	14,042.0	14,350.2	14,789.3	15,878.0	13,337.7 ± 2296.8	
February	8800.9	9282.2	9613.3	10,747.4	12,905.0	13,249.8	10,766.4 ± 1904.6	
March	10,015.8	9800.6	11,875.4	12,439.5	12,703.2	17,418.2	12,375.4 ± 2756.4	
April	8877.0	8924.5	11,509.2	12,589.8	12,493.0	15,778.5	11,695.3 ± 2598.6	
May	9591.8	10,061.8	11,783.5	12,878.5	13,897.6	14,962.1	12,195.9 ± 2122.2	
June	9543.9	9934.1	11,014.4	12,123.2	15,544.7	15,881.8	12,340.3 ± 2764.9	
July	9159.6	10,851.0	12,403.4	14,022.6	15,399.5	15,741.1	12,929.6 ± 2608.1	
August	9640.9	10,952.0	11,780.9	12,737.2	13,571.1	15,671.5	12,392.3 ± 2110.9	
September	8037.7	9701.3	9616.2	12,002.0	12,548.8	13,604.4	10,918.4 ± 2122.4	
October	8996.5	9568.2	11,972.6	12,226.9	14,578.7	14,782.3	12,020.9 ± 2423.9	
November	9439.0	10,172.6	11,146.5	12,358.8	14,937.3	15,974.4	12,338.1 ± 2626.5	
December	9542.4	9876.0	11,060.5	13,505.5	15,168.2	15,882.9	12,505.9 ± 2730.5	
Mean ± SD *	9342.3 ± 626.3	9969.0 ± 596.5	11,484.8 ± 1185.6	12,665.1 ± 962.6	14,044.7 ± 1166.7	15,402.1 ± 1122.1	12,151.4 ± 2352.3	<0.001 ^†^

Unit, million KRW; values are presented as exact number or mean ± standard deviation; * SD, standard deviation; ^†^ *p*-value < 0.05, statistically significant.

## Data Availability

Publicly available datasets were analyzed in this study. The data can be found here: https://www.data.go.kr/en/index.do (accessed on 1 December 2022).
